# President of King Edward's Hospital Fund

**Published:** 1919-05-03

**Authors:** 


### The Hospital

The Workers' Newspaper of Administrative Medicine and Institutional Life, Administration, National Insurance and Health.

No. 1717, Vol. LXVI. SATURDAY, MAY 3, 1919. PRICE TWOPENCE

### H.R.H. THE PRINCE OF WALES.

### President of King Edward's Hospital Fund.

King Edward took a great personal and continuous interest in our hospitals, which led to the establishment of King Edward's Hospital Fund. The Metropolitan hospitals were thus relieved of many financial difficulties, and greater strength was given to the voluntary hospitals of London, with much that was helpful and tended to promote the efficiency, economical improvement, and increased usefulness of these great institutions. King Edward was succeeded in the Presidency by His Majesty King George, who held office when Prince of Wales. He took an active personal interest in everything which related to the King's Fund and hospitals of all kinds, to the work of which he systematically rendered continuous personal service. Throughout his term of office many developments took place, and the speeches His Majesty made during the period he was President resulted in a large addition to the invested funds and financial strength of the King's Fund, and tended throughout to help the hospitals to improve their efficiency and to increase their funds. King George has acquired a sound knowledge of hospital administration in all departments, and His Majesty's many suggestions and practical interest have proved invaluable in many directions.

The accession to-day to the Presidency of the King's Fund of H.R.H. the Prince of Wales has been welcomed by the whole nation, for the personality of the Prince is known to the British people with the Navy and Army, who have extended to him a confidence and affection which must prove helpful and inspiring. Wherever the Prince has gone, and amongst all classes with whom he has mingled both at home and abroad, he has won golden opinions and confidence. We believe that his education from boyhood upwards has been conceived upon a plan far wiser and better calculated to bring out all that is best in his character than probably any prince the world has ever known. When thirteen he went to Osborne, two years later to Dartmouth, and subsequently was appointed a midshipman to the "Hindustan." Three years later he went to Paris as the Earl of Chester, studying French and the institutions and people of France. He was everywhere received, like his grandfather, with cordiality and interest. On his return he became a student at Magdalen College, Oxford, where he lived the life of an ordinary undergraduate in every particular. One great feature of his character is his keenness, and this was shown by the progress he made at Oxford, and by the energetic way in which he threw himself into games and sports. He showed an equal keenness in the Navy, and in everything he has been eager to learn, to gain knowledge and the power to accomplish. The more active the work, the more difficult the task, the more His Royal Highness appreciates it. Keen on the Navy, when the war broke out he became anxious to see active service. On August 8 he was gazetted a second lieutenant to the Grenadier Guards, and immediately afterwards he entered with zest on the requisite training to fit him for active service. Three months later he was promoted to lieutenant, and gazetted to Sir John French's staff at the seat of war. His record there can be gathered from the following description taken from a soldier's letter: "Our Prince is a soldier, and a man with a bigger heart than a lot who are holding back in Great Britain." The Prince also saw active service for some months in Italy, and spent nearly four years on these two fronts.

The Prince has lived a strenuous, devoted life throughout his youth and since he entered upon manhood. His Royal Highness has to-day a record which has won for him everywhere the affection and confidence of all who have had the pleasure of being associated with him at school, at college, in the Navy, the Army, and in foreign countries. It has been well said of His Royal Highness that the public loves its Prince for just so much as there has been in him of the normal, shy and natural boy, as well as for the sense of Royalty which has exalted him above the common when need was. It will never be forgotten that the Prince spent his twenty-first birthday at the seat of war. There were no public rejoicings or outward evidence of popular enthusiasm on His Royal Highness's coming of age, but never was a Royal Prince's birthday so well kept in the homes of the people, with every manifestation of genuine affection and loyalty. This affection and loyalty have grown with the years that have passed, and there is not a home throughout the Empire where these feelings do not cheer and help the people of all classes and shades of opinion.

With peace within sight His Royal Highness has commenced to take up the responsibilities which will devolve upon him as Prince of Wales. Following the example of Queen Victoria, King Edward and King George, with the members of the Royal Family, he, like them, is a strenuous upholder of the voluntary system of hospitals. This knowledge is enforced by the fact that on April 24 he accepted the presidency of St. Bartholomew's Hospital, to which office the Governors had the honour to elect him. This office was held successively by the Prince's grandfather and father, and since King George ascended the throne it has been held in abeyance with a view to the election now made. On April 25 the appointment of His Royal Highness as President of the King's Hospital Fund was announced. His acceptance of both offices has been received with enthusiasm, for it testifies to His Royal Highness's intention to follow the example of his grandfather and father with keenness and conviction. Their labours have provided for him in the field of charity a goodly heritage.

King Edward's Hospital Fund for London has proved of vast importance to the hospitals of the Metropolis and home counties, and so long as it is maintained under efficient management it cannot fail to be of invaluable assistance to some of our greatest voluntary institutions. This Fund has had a quickening influence for good in the administration and management of all institutions to which grants have been made. Nowhere will the Prince receive a warmer welcome than in the field of charity, and nowhere, we are confident, can his services and personal influence be more beneficial or greater.

